# Supplementary material for: Empirical methods for controlling false positives and estimating confidence in ChIP-Seq peaks
Source: BMC Bioinformatics. 2008 Dec 5;9:523. doi: 10.1186/1471-2105-9-523 (PMC2628906; doi:10.1186/1471-2105-9-523)
Supplement: Additional File 1 — A variety of html documents from the USeq web site detailing the available applications, their best usage, output file type descriptions, command line menus, etc. [file 1471-2105-9-523-S1.zip › USeqUserGuides/JavaDoc.zip!/JavaDoc/selex/SeqFiles.html]

SeqFiles


|  |  |  |
| --- | --- | --- |
| **Overview** \| **Package** \| **Class** \| **Use** \| **Tree** \| **Deprecated** \| **Index** \| **Help** | |  |
| **PREV CLASS**   **NEXT CLASS** | **FRAMES**    **NO FRAMES**     **All Classes** |  |
| SUMMARY: NESTED \| FIELD \| CONSTR \| METHOD | DETAIL: FIELD \| CONSTR \| METHOD |  |


---


## selex Class SeqFiles

```
java.lang.Object
  selex.SeqFiles
```

---

``` public class SeqFiles extends java.lang.Object ```

Continer for raw sequence information, seqFile, qualFile, and the actual reads from each.

---

**Constructor Summary**

`SeqFiles(java.lang.String aSeqFile,
java.lang.String aQualFile,
SelexParams SP)`


| **Method Summary** | |
| --- | --- |
| `static java.lang.String` | `changeQuality(java.lang.String qual, int index, int diff)`             Modifies a quality file, adding or subtracting scores. |
| `int` | `getNumReads()` |
| `java.lang.String` | `getQualFile()` |
| `java.lang.String` | `getSeqFile()` |
| `SeqRead[]` | `getSeqReads()` |
| `static void` | `modifySequenceEnds(java.lang.String[] seqs, java.lang.String[] quals, java.lang.String leftSideMatch, java.lang.String leftSideReplace, java.lang.String rightSideMatch, java.lang.String rightSideReplace)`             Modifies the left side and right side of Sequences also adds (100's) or subtracts quanilty scores to match. |
| `void` | `printSeqFiles()` |
| `static java.lang.String` | `stringArrayListToString(java.util.ArrayList stringAL, java.lang.String separator)`             Returns a String separated by the separator given an ArrayList of String. |
| `static java.util.ArrayList` | `stringArrayToArrayList(java.lang.String[] s)`             Returns an ArrayList of String given a String[] |

**Methods inherited from class java.lang.Object**

`equals, getClass, hashCode, notify, notifyAll, toString, wait, wait, wait`

**Constructor Detail**

### SeqFiles

```
public SeqFiles(java.lang.String aSeqFile,
                java.lang.String aQualFile,
                SelexParams SP)
```


**Method Detail**

### getSeqFile

```
public java.lang.String getSeqFile()
```

---


### getQualFile

```
public java.lang.String getQualFile()
```

---


### getNumReads

```
public int getNumReads()
```

---


### getSeqReads

```
public SeqRead[] getSeqReads()
```

---


### printSeqFiles

```
public void printSeqFiles()
```

---


### modifySequenceEnds

```
public static void modifySequenceEnds(java.lang.String[] seqs,
                                      java.lang.String[] quals,
                                      java.lang.String leftSideMatch,
                                      java.lang.String leftSideReplace,
                                      java.lang.String rightSideMatch,
                                      java.lang.String rightSideReplace)
```

:   Modifies the left side and right side of Sequences also adds (100's) or subtracts quanilty scores to match.
    Will modify the first occurance on the left, and the last occurance on the right.
    Usefull for fixing blunted sequence ends for Selex parsing.
    Case insensitive.

---


### changeQuality

```
public static java.lang.String changeQuality(java.lang.String qual,
                                             int index,
                                             int diff)
```

:   Modifies a quality file, adding or subtracting scores.

---


### stringArrayToArrayList

```
public static java.util.ArrayList stringArrayToArrayList(java.lang.String[] s)
```

:   Returns an ArrayList of String given a String[]

---


### stringArrayListToString

```
public static java.lang.String stringArrayListToString(java.util.ArrayList stringAL,
                                                       java.lang.String separator)
```

:   Returns a String separated by the separator given an ArrayList of String.


---


|  |  |  |
| --- | --- | --- |
| **Overview** \| **Package** \| **Class** \| **Use** \| **Tree** \| **Deprecated** \| **Index** \| **Help** | |  |
| **PREV CLASS**   **NEXT CLASS** | **FRAMES**    **NO FRAMES**     **All Classes** |  |
| SUMMARY: NESTED \| FIELD \| CONSTR \| METHOD | DETAIL: FIELD \| CONSTR \| METHOD |  |


---
